# Supplementary material for: Engram-specific transcriptome profiling of contextual memory consolidation
Source: Nat Commun. 2019 May 20;10:2232. doi: 10.1038/s41467-019-09960-x (PMC6527697; doi:10.1038/s41467-019-09960-x)
Supplement: Supplementary file 1 — Supplementary Information [file 41467_2019_9960_MOESM1_ESM.pdf]

Supplementary Information

Engram-specific transcriptome profiling of contextual memory consolidation

Rao-Ruiz et. al.

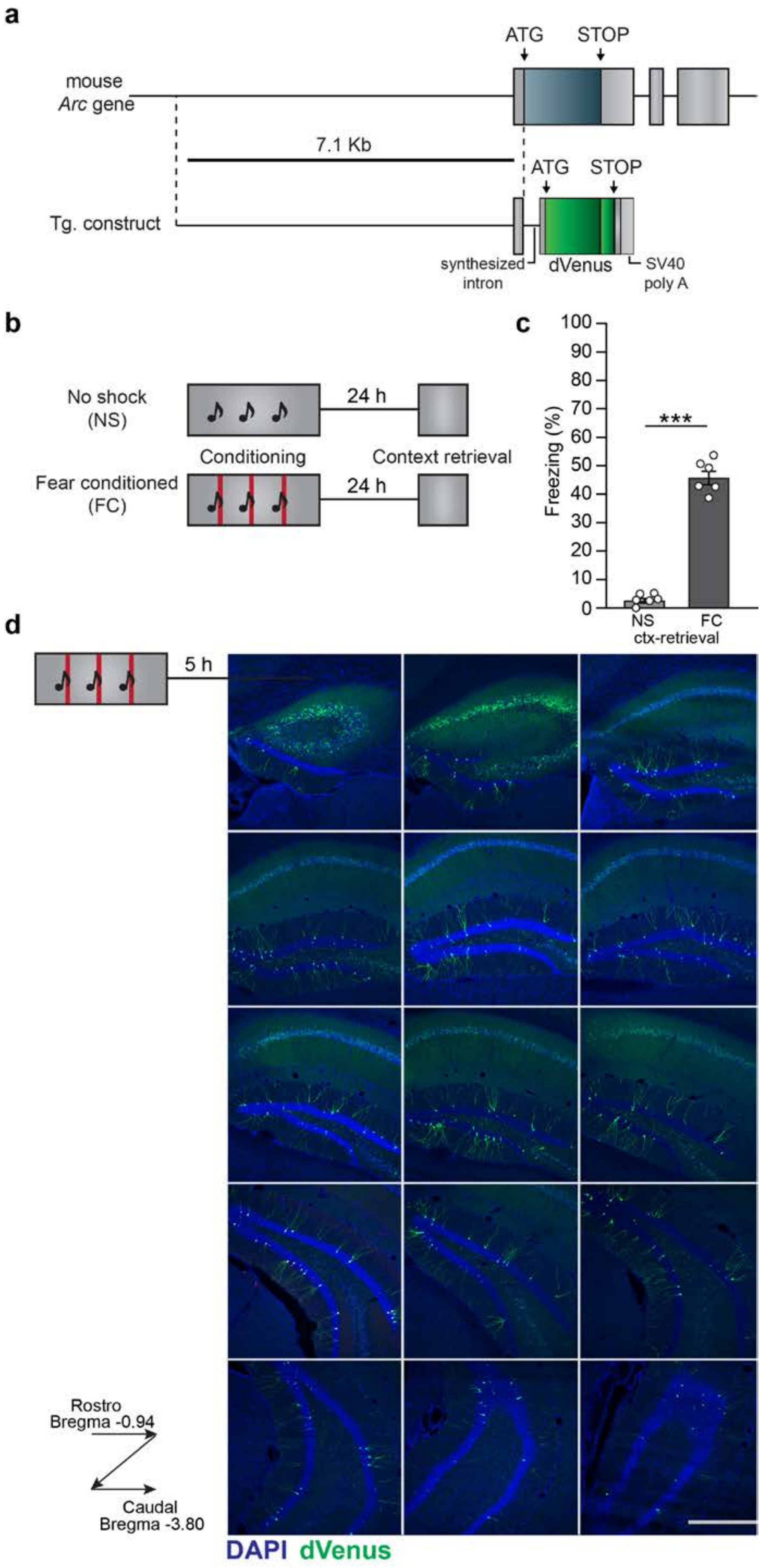
a
ATG
STOP
mouse
Arc gene
7.1 Kb
ATG
STOP
Tg. construct
synthesized intron
dVenus
SV40 poly A
b
No shock (NS)
24 h
Conditioning
Context retrieval
Fear conditioned (FC)
24 h
c
Freezing (%)
0
10
20
30
40
50
60
70
80
90
100
***
NS
FC
ctx-retrieval
d
5 h
Rostro
Bregma -0.94
Caudal
Bregma -3.80
DAPI dVenus

Supplementary Figure 1. Fear conditioning induces a robust contextual fear memory and the recruitment of a sparse population of *Arc*::dVenus$^+$ neurons in the DG

(a) A 7.1 kb fragment containing the 5′-upstream region of the mouse *Arc* gene drives the expression of the *destabilized Venus* fluorescent reporter in the *Arc*::dVenus construct used to generate transgenic mice [29]. A synthetic intron and SV40 polyadenylation signal enhance the level of gene expression.

(b) *Arc*::dVenus transgenic mice were exposed to the conditioning chamber, either in the absence of foot-shocks (No shock, NS controls, n=6) or with 3 tone-shock pairings (fear conditioned, FC, n=6). Contextual fear memory was tested 24h later in the conditioning context.

(c) FC mice exhibit robust fear memory, while NS mice receiving no shock exhibit low levels of freezing. Analysis of variance: $F(1,11)=303.6$, $P=2.2 \times 10^{-9}$. ***P<0.001. Data presented as mean ± SEM. Source data are provided as a Source Data file.

(d) Sparse expression of dVenus in DG granule cells 5h after fear conditioning. Scale bar: 500µm. dVenus native fluorescence (green) and DAPI (blue). Bregma coordinates are based on the Mouse brain atlas[23].

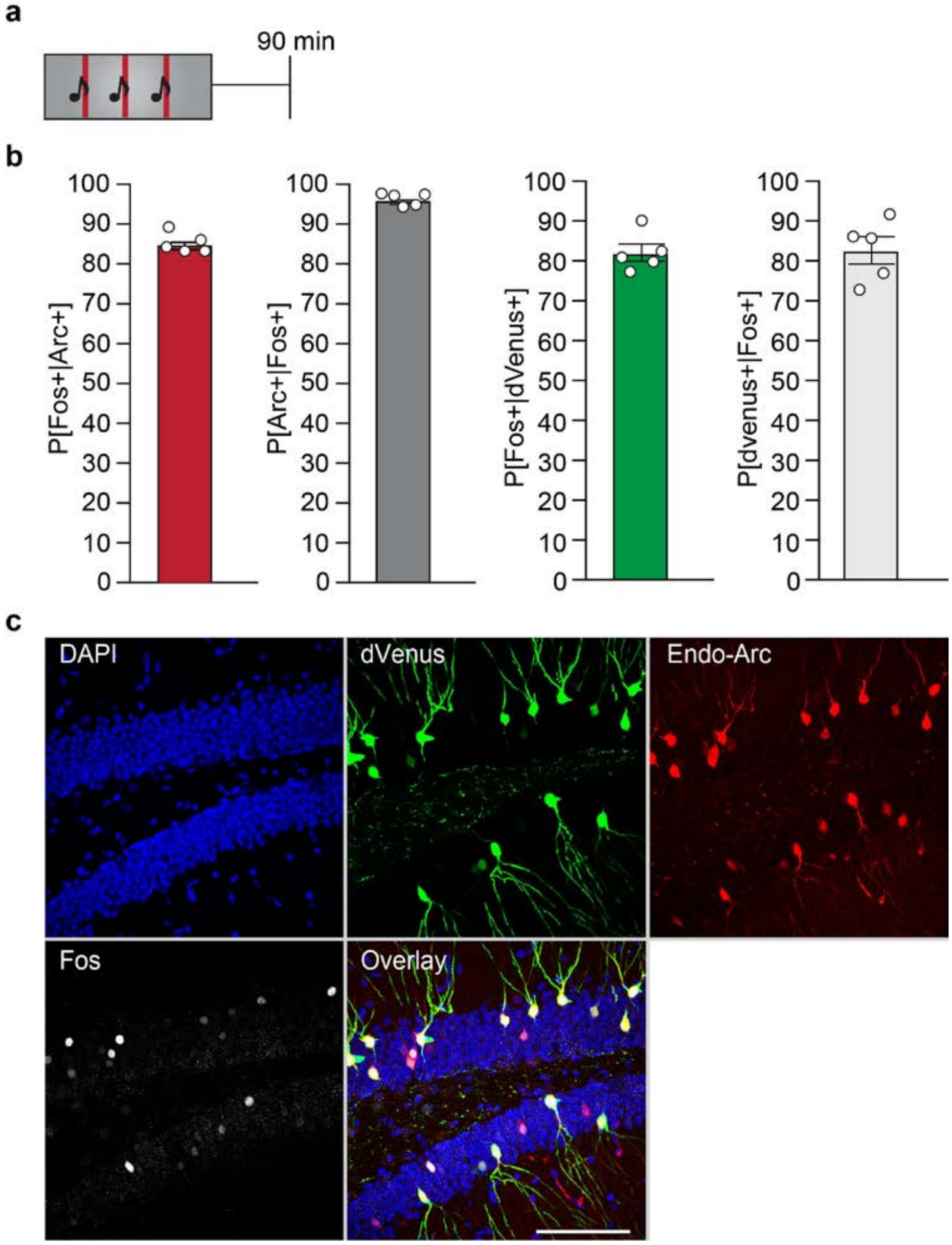

Supplementary Figure 2. Cellular co-localization of the immediate early genes Arc and Fos

(a) Experimental setup. *Arc*::dVenus mice (n=6) were fear conditioned and sacrificed 90 min later to perform co-labelling experiments for Arc and Fos.

(b) 85.24% of Arc$^+$ cells also expressed Fos (P[Fos$^+$|Arc$^+$]), while 96.32% of Fos$^+$ cells also expressed Arc (P[Arc$^+$|Fos$^+$]). 82.91% of dVenus$^+$ cells also expressed Fos (P[Fos$^+$|dVenus$^+$]), while 82.7 Fos$^+$ cells also expressed dVenus. Data presented as mean ± SEM. Source data are provided as a Source Data file.

(c) Representative images of the DG showing the expression of DAPI (blue), dVenus (green), endogenous Arc (red) and Fos (greyscale). Scale bar: 100µm.

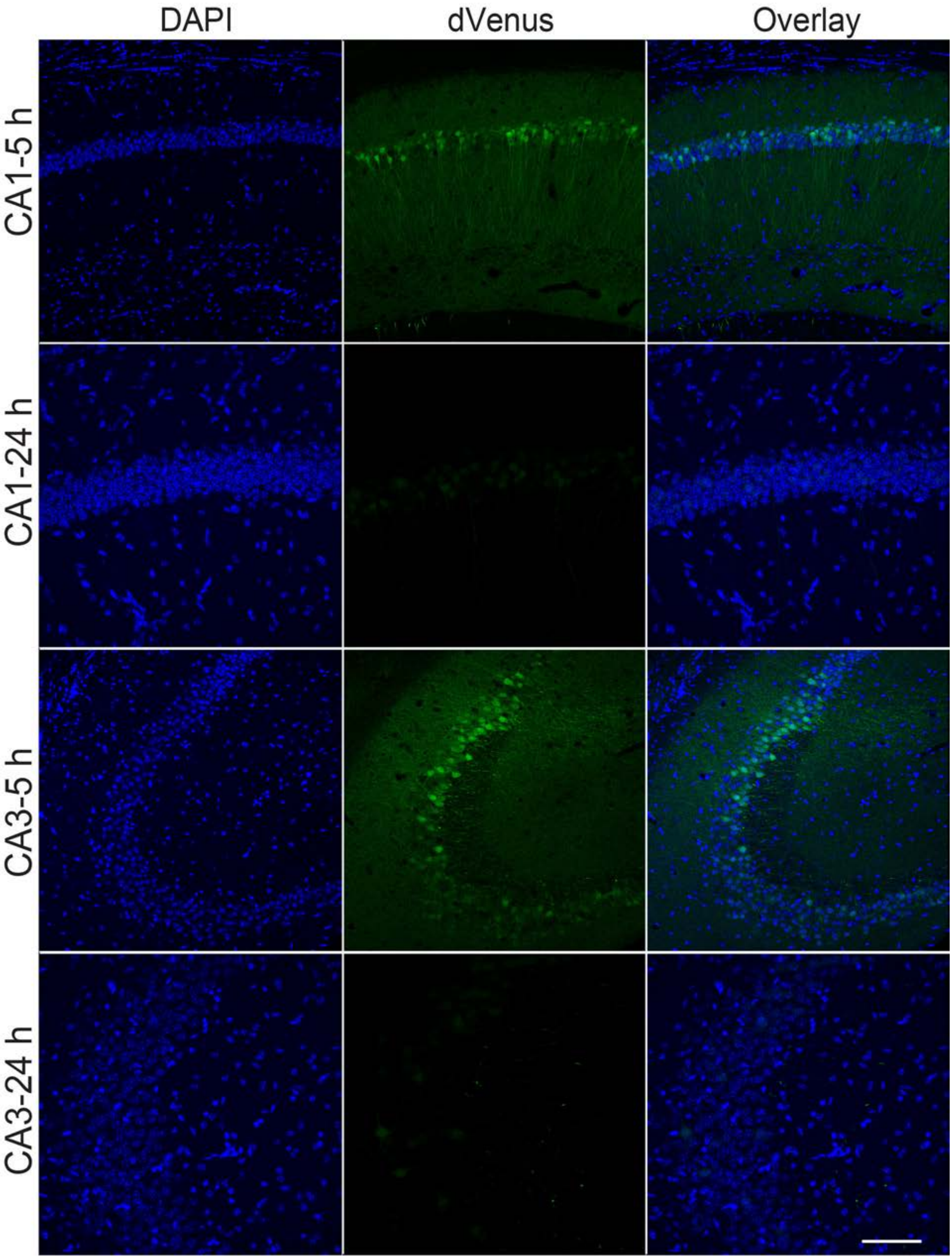
DAPI
dVenus
Overlay
CA1-5 h
CA1-24 h
CA3-5 h
CA3-24 h

Supplementary Figure 3. dVenus is expressed at 5h, but not 24h after fear conditioning in the CA1 and CA3 subfields of the hippocampus

Arc::dVenus mice were fear conditioned and sacrificed at 5h or 24h after conditioning. dVenus$^+$ cells were observed in the CA1 and CA3 subfields of the hippocampus 5 h after conditioning, but not at 24 h. Blue: Cell nuclei stained with DAPI, Green: dVenus native fluorescence. Scale bar: 200µm.

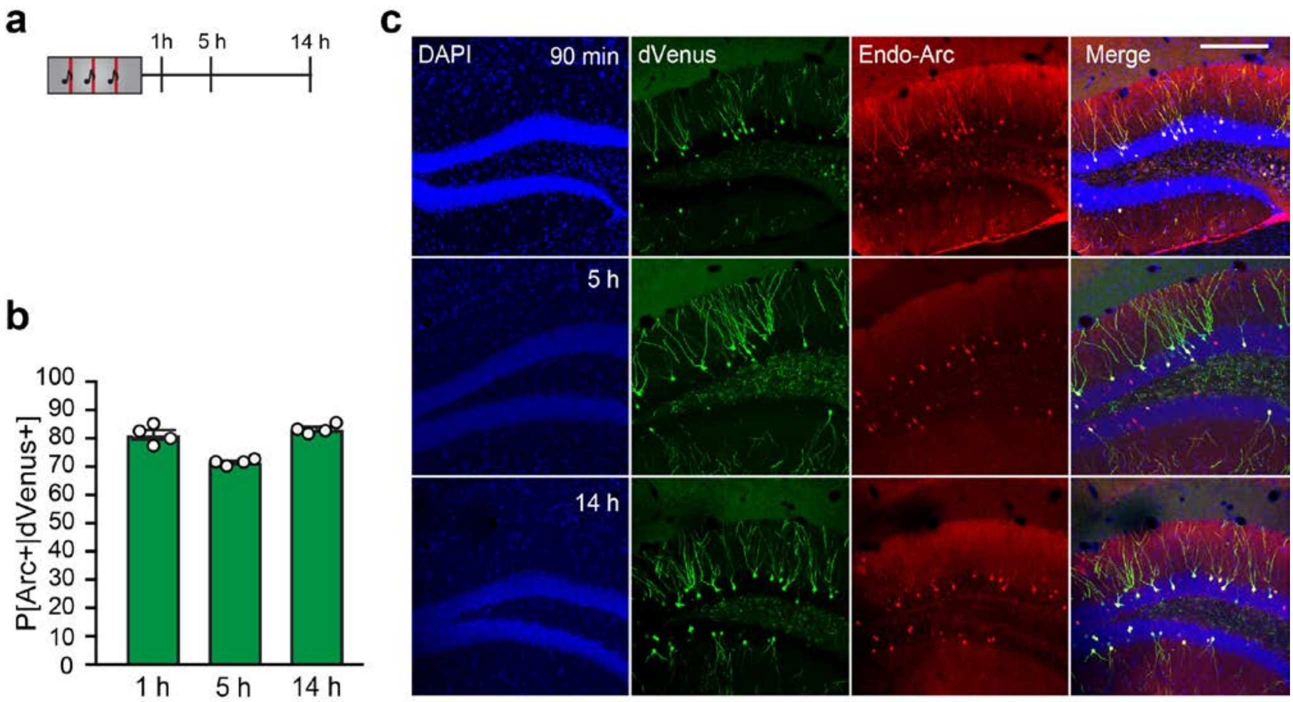

Supplementary Figure 4: Temporal expression profile of activity-dependent, sustained expression of endogenous *Arc* in DG granule cells after fear conditioning

(a) Experimental setup. *Arc*::dVenus mice were fear conditioned and the co-localization of dVenus and endogenous Arc protein was measured in DG granule cells at successive time-points.

(b) Percentage of dVenus^{+} cells in the DG that also express endogenous Arc 1 h (n=4), 5 h (n=4) and 14 h (n=4) after fear conditioning. Data is presented as mean ± SEM. Source data are provided as a Source Data file.

(i) Representative images demonstrating co-labeling of endogenous Arc and dVenus. Scale bar: 100µm

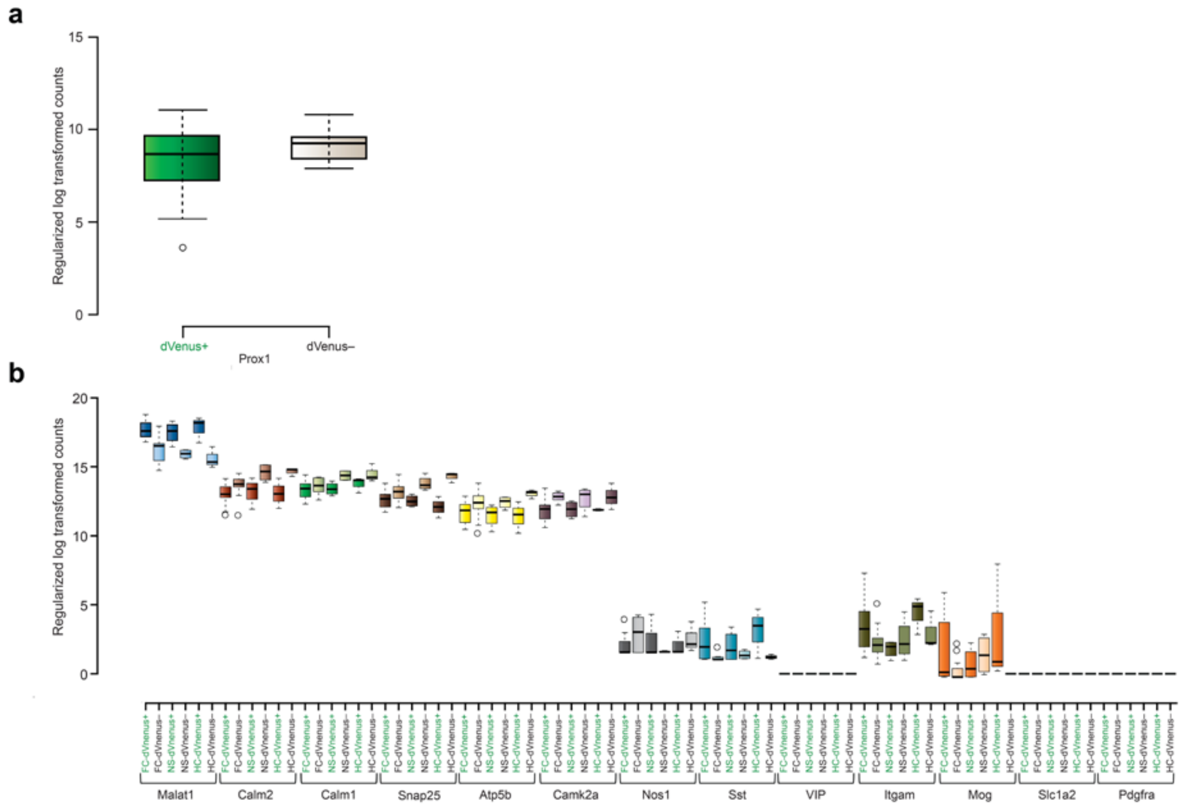

Supplementary Figure 5. Normalized gene counts for granule cell transcriptional identity

(a) Regularized log counts of the DG granule cell marker *Prox1* [37] in dVenus^{+} and dVenus^{-} cells from the fear conditioned (FC) group confirmed that transcriptome analysis was performed selectively in DG granule cells.

(b) The genes *Malat1*, *Calm2*, *Calm1*, *Snap25* and *Atp5b* that have previously been identified as having the highest expression in dorsal DG granule cells[14] were also robustly expressed. Furthermore all samples showed high levels of the forebrain principle neuronal marker *Camk2a*. Inhibitory neuronal markers [38] *Nos1*, *Sst* and *VIP*, as well as markers for non-neuronal cells [38] *Itgam* (microglia), *Mog* (oligodendrocytes), *Slc1a2*/*Glt1* (astrocytes) and *Pdgfra* (oligodendrocyte precursor cells) showed low or undetectable expression. HC: home cage controls, NS: no shock controls, FC: fear conditioned.

Source data are provided as a Source Data file.

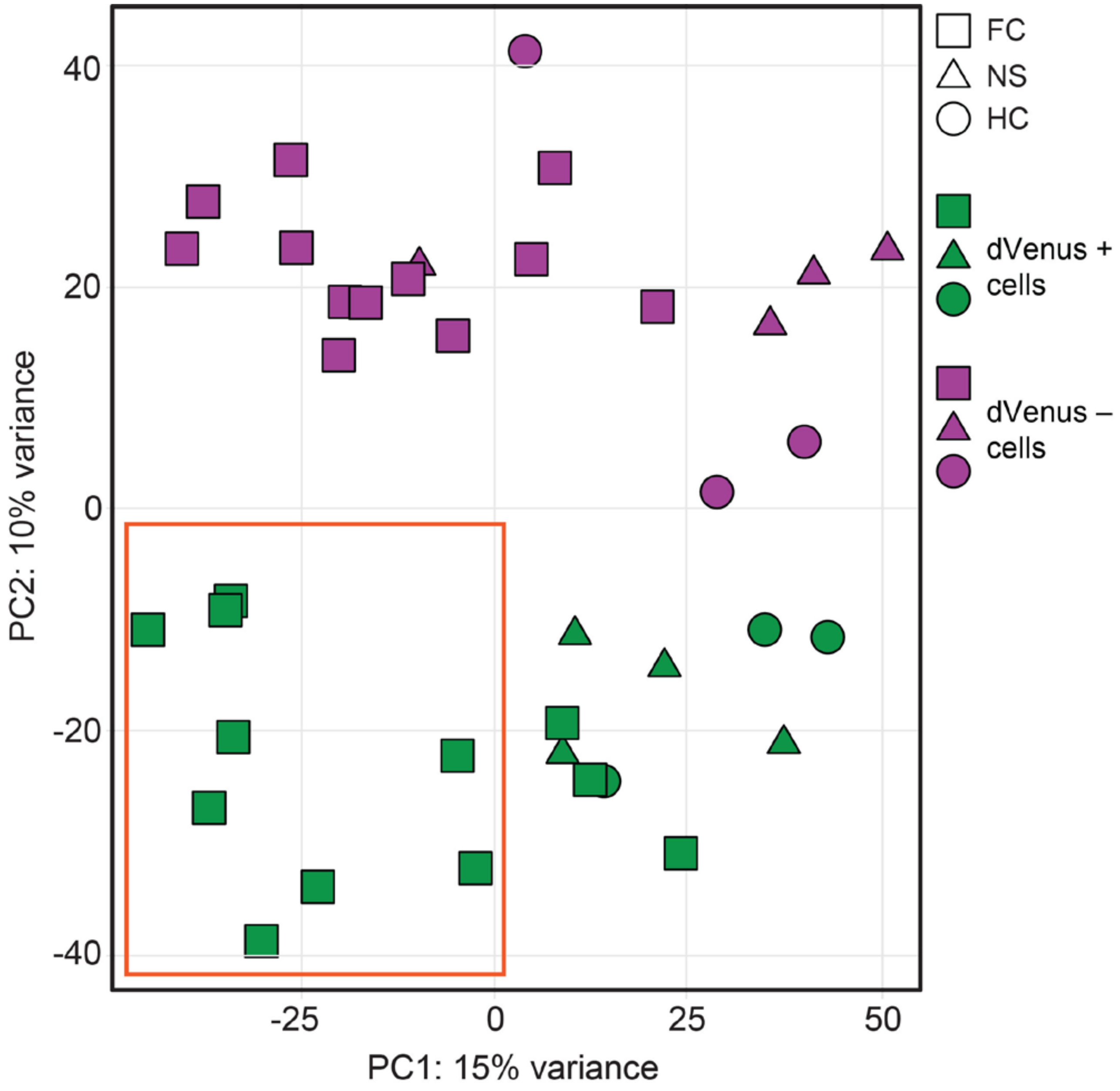

Supplementary Figure 6. Sample-to-sample principal component analysis of the top 500 differentially regulated genes.

PC1 score distinguishes dVenus^{+} cells from dVenus^{-} cells across all experimental groups, while PC2 separates dVenus^{+} cells by experimental condition with dVenus^{+} cells from the fear conditioned group (FC) segregating from dVenus^{+} cells of naïve home-cage controls (HC) and no-shock controls (NS). Orange rectangle delineates the corresponding PC1/PC2 isolated quadrant.

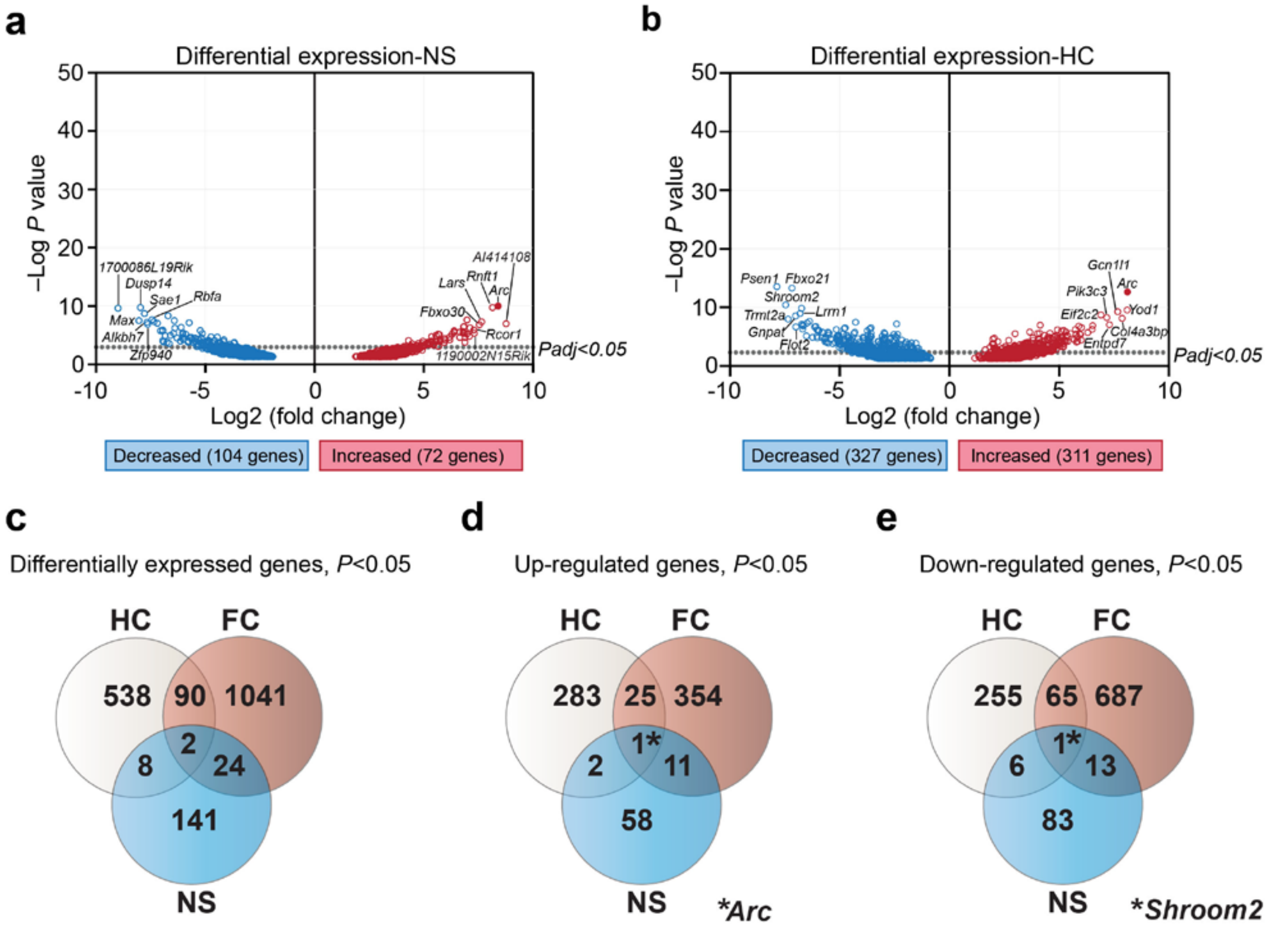

Supplementary Figure 7. Differential gene expression analysis of dVenus$^+$ *vs.* dVenus$^-$ cells.

(a, b) Differential expression results between dVenus$^+$ cells and dVenus$^-$ cells cells for all genes from the **(a)** No-shock (NS) and **(b)** home-cage (HC) experimental groups, with a raw P<0.05. Dotted line indicates P_{adj}<0.05 (FDR corrected). Genes that are upregulated in dVenus$^+$ cells are in red, and genes that are down regulated in dVenus$^+$ cells are in blue. The top 7 up and down regulated genes along with the total number of regulated genes with P_{adj}<0.05 are labeled.

(c-e) Venn diagrams demonstrating the number of significant differentially expressed genes (dVenus$^+$ *vs.* dVenus$^-$) exclusive to each experimental condition and the number of significantly regulated genes that overlap between the different experimental groups **(c)** displays all genes while **(d)** shows up regulated genes and **(e)** down regulated genes. Only 2 genes were differentially expressed across all three groups, with *Arc* being up regulated and *Shroom2* being down regulated in dVenus$^+$ DG granule cells.

HC: Home-cage controls, NS: No-shock controls, FC: Fear conditioned.

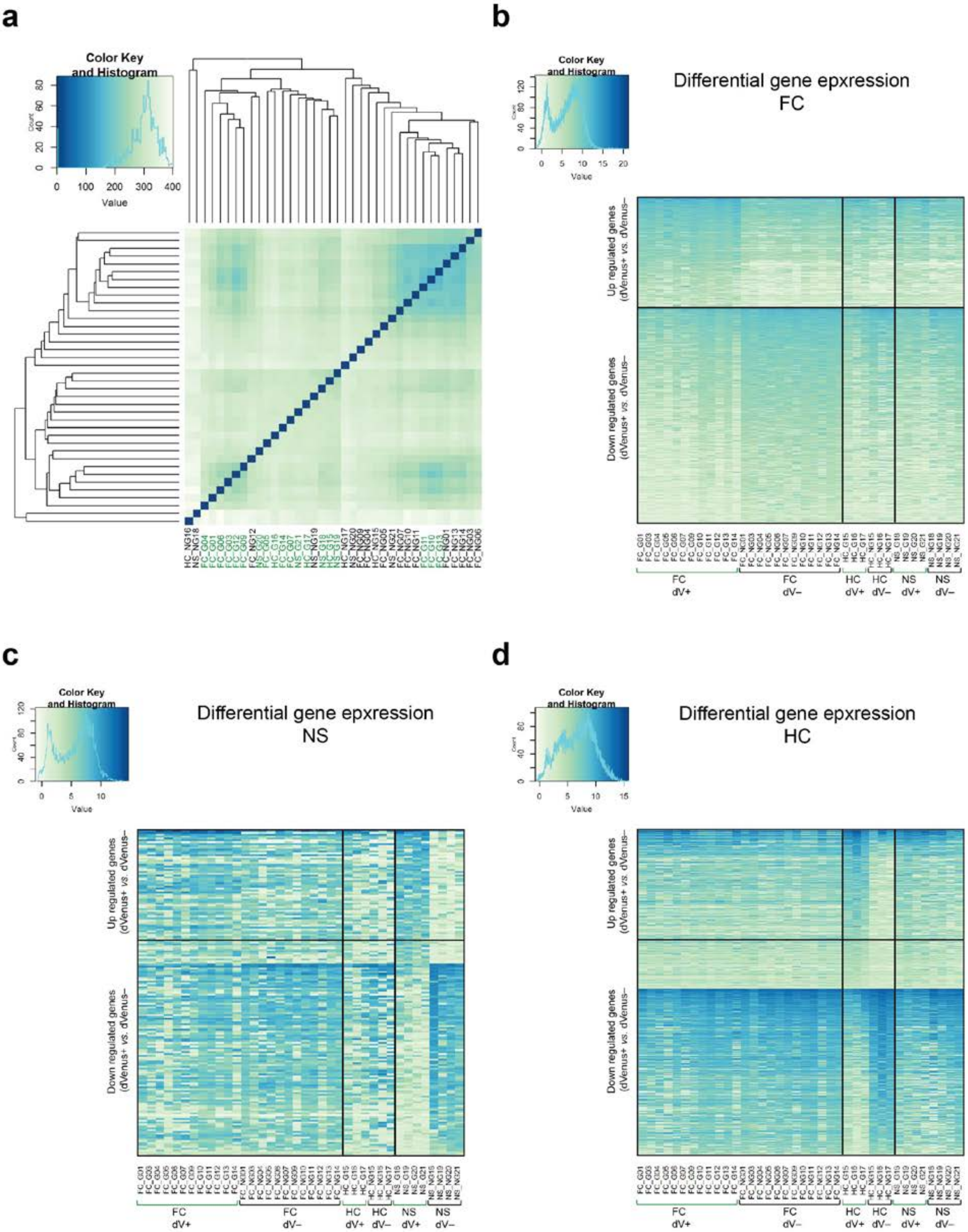
a
Color Key and Histogram
Count
Value
b
Color Key and Histogram
Value
Differential gene epxression
FC
Up regulated genes (dVenus+ vs. dVenus−
Down regulated genes (dVenus+ vs. dVenus−
FC dV+
FC dV−
HC dV+
HC dV−
NS dV+
NS dV−
c
Color Key and Histogram
Value
Differential gene epxression
NS
Up regulated genes (dVenus+ vs. dVenus−
Down regulated genes (dVenus+ vs. dVenus−
FC dV+
FC dV−
HC dV+
HC dV−
NS dV+
NS dV−
d
Color Key and Histogram
Value
Differential gene epxression
HC
Up regulated genes (dVenus+ vs. dVenus−
Down regulated genes (dVenus+ vs. dVenus−
FC dV+
FC dV−
HC dV+
HC dV−
NS dV+
NS dV−

Supplementary Figure 8. Variability between libraries

(a) Sample to sample distances were calculated as Euclidian distances between regularized log-transformed expression vectors for each sample. Dendrograms were computed by complete hierarchical clustering of the distances. FC: Fear conditioned, NS: No shock, HC: Home cage, G: green, $dVenus^{+}$, NG: non-green, $dVenus^{-}$.

(b-d) Heat map of expression values for the genes differentially expressed between dVenus+ and dVenus- for the [HC/FC/NS] samples. Up regulated genes are in the top half of each heat plot while down regulated genes are in the bottom half, with both sets being ordered by decreasing order of mean expression. FC: Fear conditioned, NS: No shock, HC: Home cage, dV: dVenus.

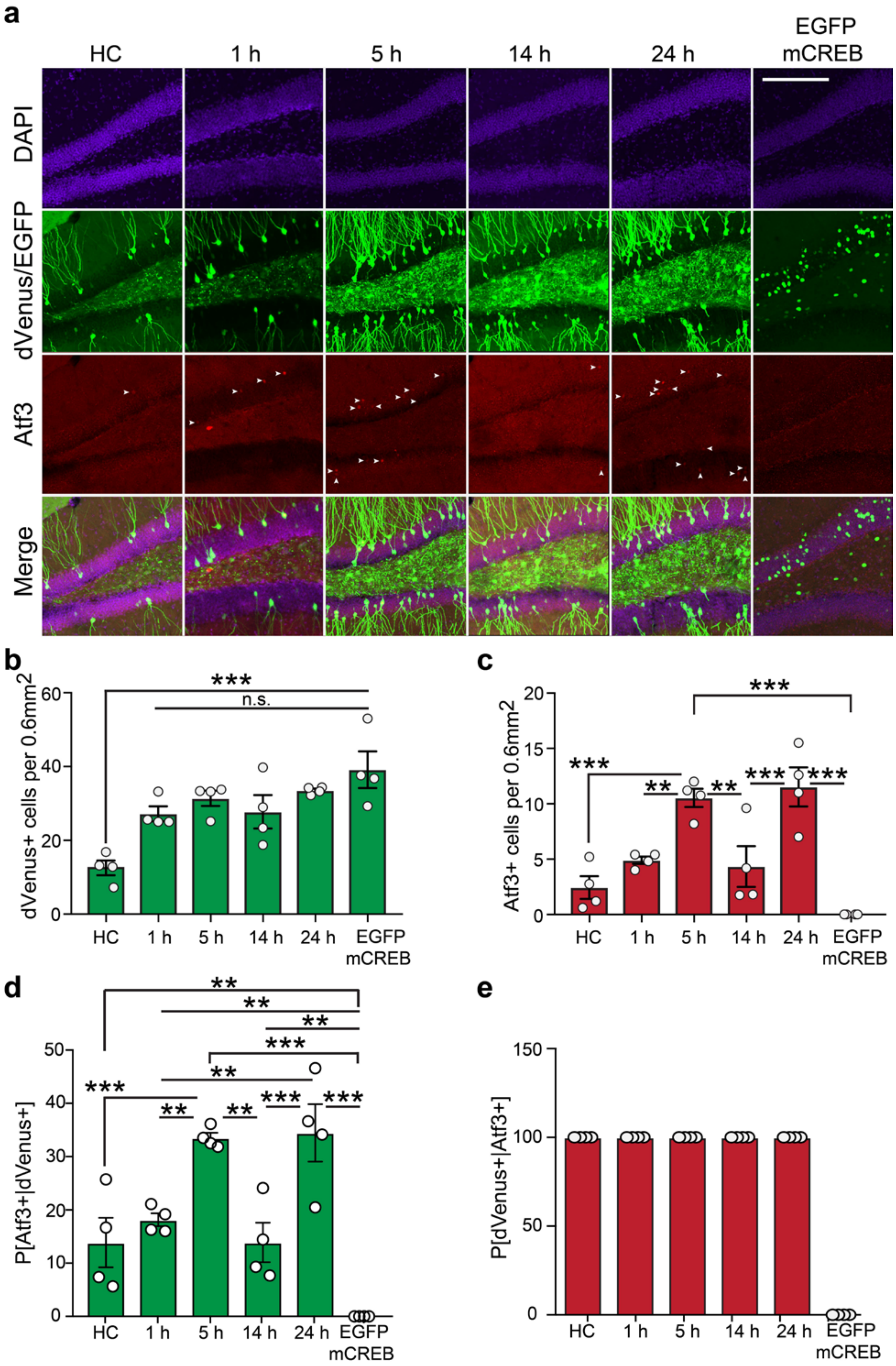
a
HC
1 h
5 h
14 h
24 h
EGFP mCREB
DAPI
dVenus/EGFP
Atf3
Merge
b
dVenus+ cells per 0.6mm2
***
n.s.
HC
1 h
5 h
14 h
24 h
EGFP mCREB
c
Atf3+ cells per 0.6mm2
***
**
d
P[Atf3+|dVenus+]
e
P[dVenus+|Atf3+]

Supplementary Figure 9. Dynamic temporal expression profile of Atf3 after fear conditioning reveals an increase of Atf3+ cells 5 h and 24 h after conditioning, and a complete loss of Atf3 in engram cells expressing mCREB.

(a) Representative images of the DG from fear conditioned mice at each successive time-point after fear conditioning. Red arrows indicate $Atf3^+$ cells in the DG granule cell layer. Scale bar: 200 µm.

(b) Number of $dVenus^+$ cells per $0.6mm^2$ section in the DG, at specific time-points after fear conditioning (n=4 mice per time-point). Analysis of variance: effect of training history over baseline (HC): $F_{(1,23)}$=17.316, P=4.1 x10^{-4} .

(c) Number of $Atf3^+$ cells per $0.6mm^2$ section in the DG, at specific time-points after fear conditioning. Effect of training history over baseline (HC): $F_{(1,23)}$=2.310, P=0.002; *post hoc* LSD: HC *vs*. 5h: P=0.002, HC *vs.* 24h: P=14.5x10^{-4}, 1 h *vs.* 5 h: P=0.034, 1 h *vs.* 14 h: P=0.009, 5 h *vs.* 14 h: P=0.017, 5 h *vs.* EGFP-mCREB: P=7.7x10^{-5}, 14 h *vs.* 24 h: P=0.0048, 24 h *vs.* EGFP-mCREB: P=2.5x10^{-5}.

(d) Percentage of $dVenus^+$ cells in the DG that also express endogenous Atf3 (n=4 mice per time-point). Analysis of variance: $F_{(5,23)}$=15.6, P= 5x10^{-6}; *post hoc* LSD: HC *vs*. 5h: P= 5.7x10^{-4}, HC *vs.* 24h: P=3.7x10^{-4}, HC *vs.* EGFP-mCREB: P=0.009, 1 h *vs.* 5 h: P=0.004, 1 h *vs.* 24 h: P=0.003, 1 h *vs.* EGFP-mCREB: P=0.001, 5 h *vs.* 14 h: P=0.001, 5 h *vs.* EGFP-mCREB: P=1.0x10^{-6}, 14 h *vs.* 24 h: P=3.7x10^{-4}, 14 h *vs.* EGFP-mCREB: P=0.009, 24 h *vs.* EGFP-mCREB: P=8.5x10^{-7}.

(e) Percentage of $Atf3^+$ cells in the DG that also express dVenus (n=4 mice per time-point).

*P<0.05, **P<0.01, ***P<0.001. Data is presented as mean ± SEM.

Source data are provided as a Source Data file.

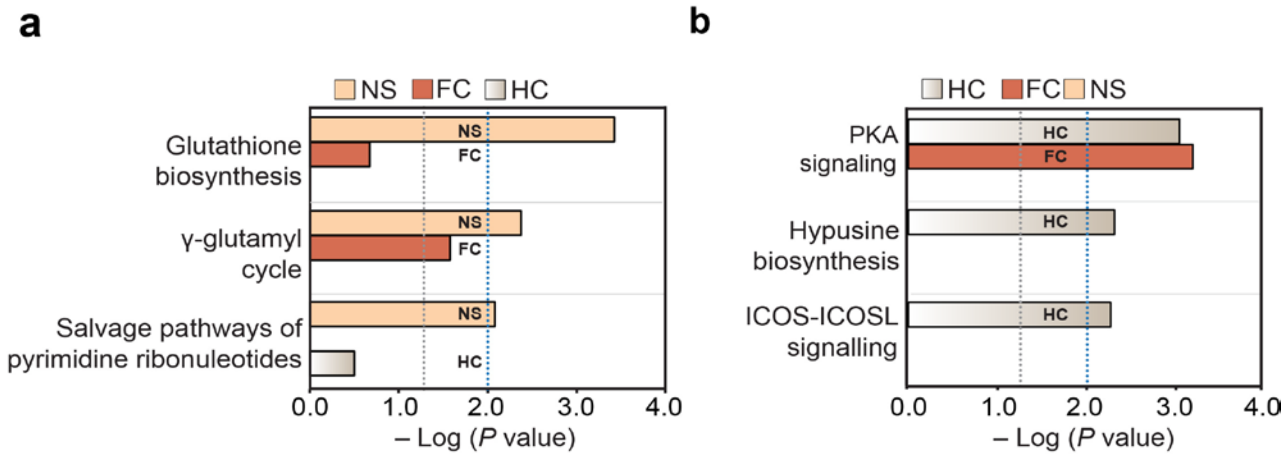

Supplementary Figure 10. Ingenuity pathway analysis of differentially expressed genes in the NS and HC groups.

(a) Functional pathway enrichment with $P < 0.01$ of differentially expressed genes in the NS group. The enrichment of these pathways in the FC and HC groups is plotted alongside the NS group. Grey dotted line indicates significance threshold set at $-\log_{10} P > 1.3$ ($P < 0.05$, Fisher's Exact Test), and blue dotted line indicates significance threshold set at $-\log_{10} P > 2$ ($P < 0.01$, Fisher's Exact Test). NS: No-shock controls, FC: Fear conditioned and HC: Home-cage controls.

(b) Functional pathway enrichment with $P < 0.01$ of differentially expressed genes in the HC group. The enrichment of these pathways in the FC and NS groups is plotted alongside the HC group. Grey dotted line indicates significance threshold set at $-\log_{10} P > 1.3$ ($P < 0.05$, Fisher's Exact Test), and blue dotted line indicates significance threshold set at $-\log_{10} P > 2$ ($P < 0.01$, Fisher's Exact Test). HC: Home-cage controls, FC: Fear conditioned, NS: No-shock controls.

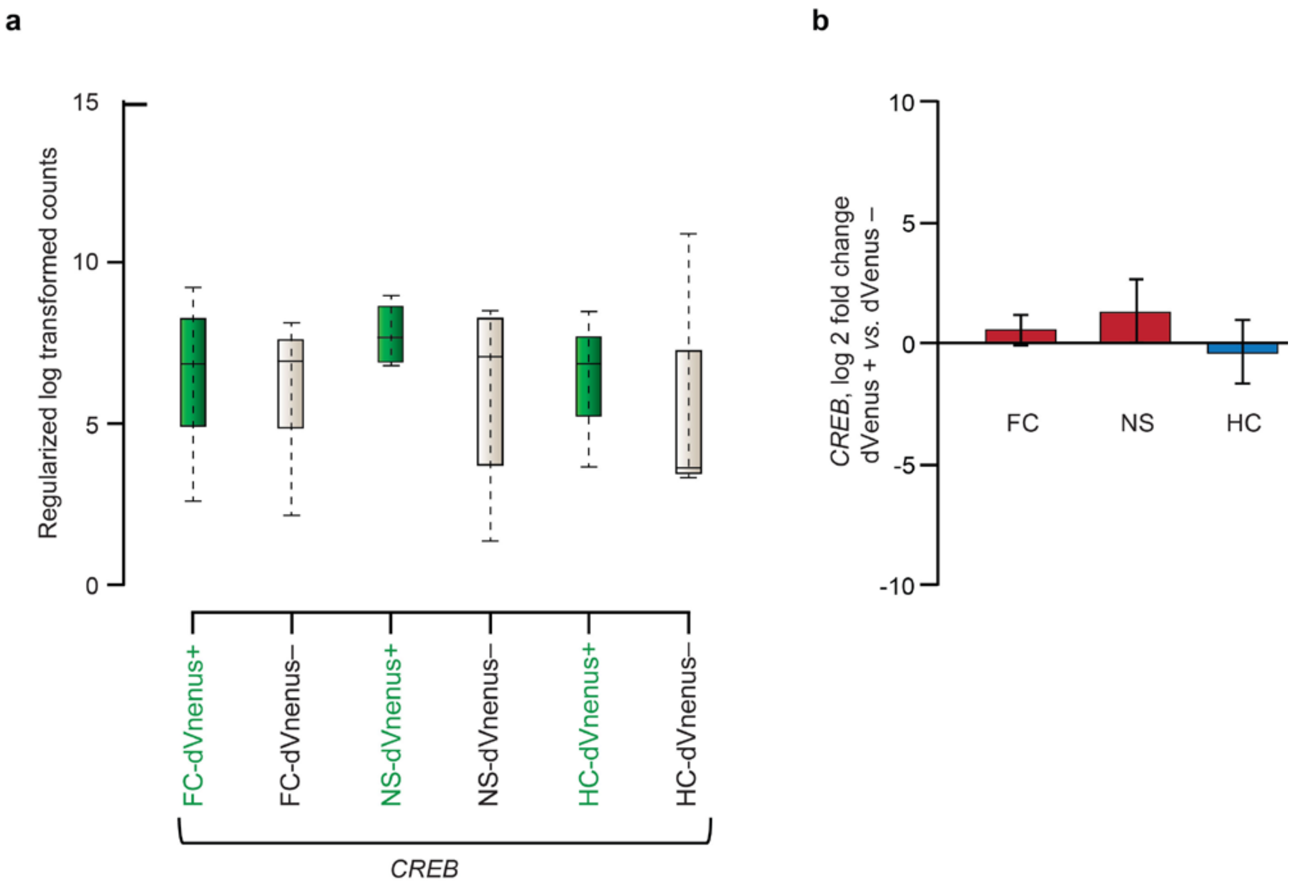

Supplementary Figure 11. Normalized gene counts and differential expression of the gene *CREB* in the Fear conditioned (FC), no-shock (NS), and home-cage (HC) groups.

(a) Regularized log counts of *CREB* in dVenus^{+} and dVenus^{-} cells from the fear conditioned (FC), no-shock (NS) and home-cage (HC) groups. Source data are provided as a Source Data file.

(b) Differential analysis of *CREB* expression revealed no significant regulation between dVenus^{+} and dVenus^{-} cells in the FC (P=0.70), NS (P=0.79) or HC (P=0.90) groups. Values are given as $\log_2$-fold change. Data are presented as mean ± SEM.

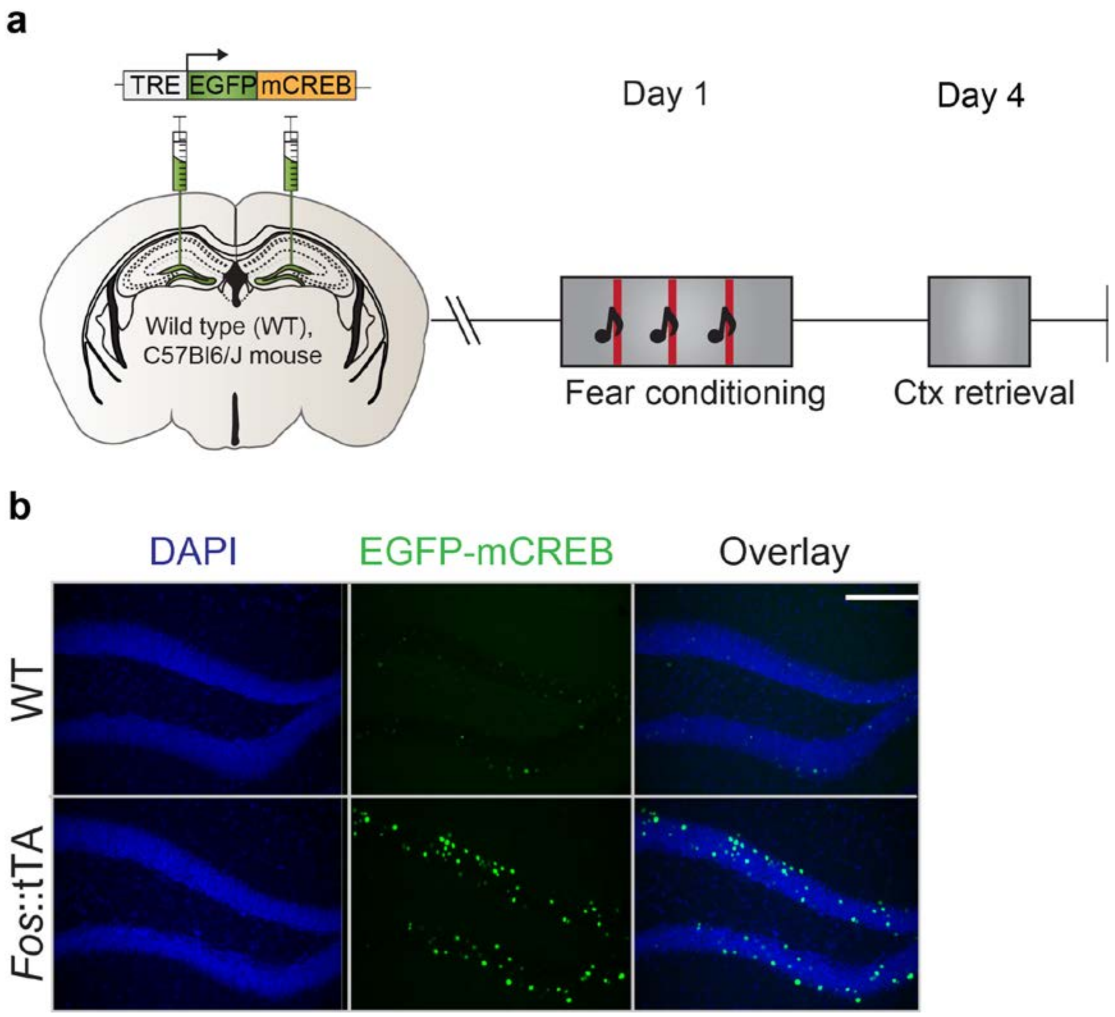

Supplementary Figure 12. Doxycycline inducible expression of mCREB is absent in WT animals

(a) Experimental setup. Wild type (WT) mice (n=4) injected with the AAV5-TRE::EGFP-mCREB virus were fear conditioned and contextual fear memory was tested 72 h later. Animals were sacrificed 90 min after the context retrieval test.

(b) Representative images of the DG from WT and *Fos*::tTA mice confirms that *Fos* promotor-driven doxycycline-controlled expression of EGFP-mCREB requires the *Fos*::tTA transgene. EGFP-mCREB (green) and DAPI (blue). Scale bar: 200μm.

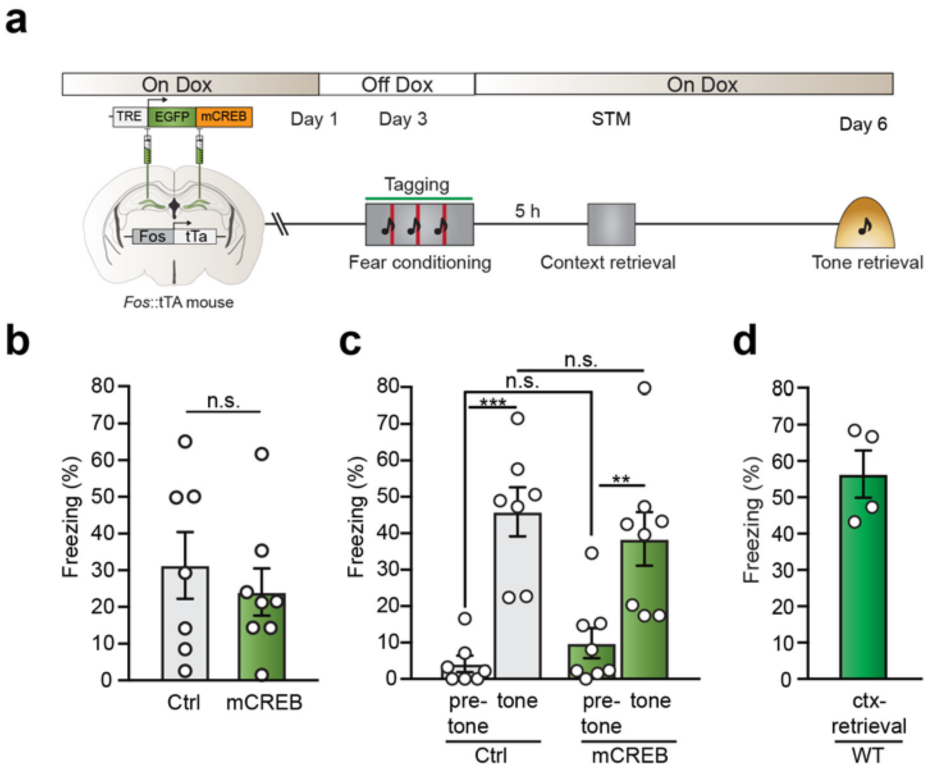

Supplementary Figure 13. Interfering with the CREB network has no effect on short-term contextual fear memory or long-term auditory fear memory

(a) Experimental design. *Fos*::tTA mice injected with AAV5-TRE::EGFP-mCREB (n=7) or AAV5-TRE::mCherry (n=8) targeting the DG were removed from Doxycycline and fear conditioned. Animals were placed back on Dox immediately after training and tested for short-term contextual memory 5 h later and long-term auditory fear memory 72 h after training.

(b) Freezing levels (%) during the contextual short-term memory test (5h post-training). Analysis of variance: Control vs. mCREB: $F_{(1,14)}$=0.4, P=0.52.

(c) Freezing levels (%) in a novel context for long-term auditory fear memory (72h post-training). Analysis of variance, Pre-tone vs. post-tone: Control: $F_{(1,12)}$=28.6, P=0.0002, mCREB: $F_{(1,15)}$= 11.5, P=0.004, Control vs. mCREB (pre-tone): $F_{(1,14)}$=1.3, P=0.27, Control vs. mCREB (tone): $F_{(1,14)}$=0.5, P=0.48.

(d) WT animals injected with AAV5-TRE::EGFP-mCREB do not show a contextual memory deficit 72 h after conditioning, consistent with the specificity of the induction of EGFP-mCREB in *Fos*::tTA transgenic mice.

n.s.: not significant, **P<0.01, ***P<0.001. Data is presented as mean ± SEM.

Source data are provided as a Source Data file.

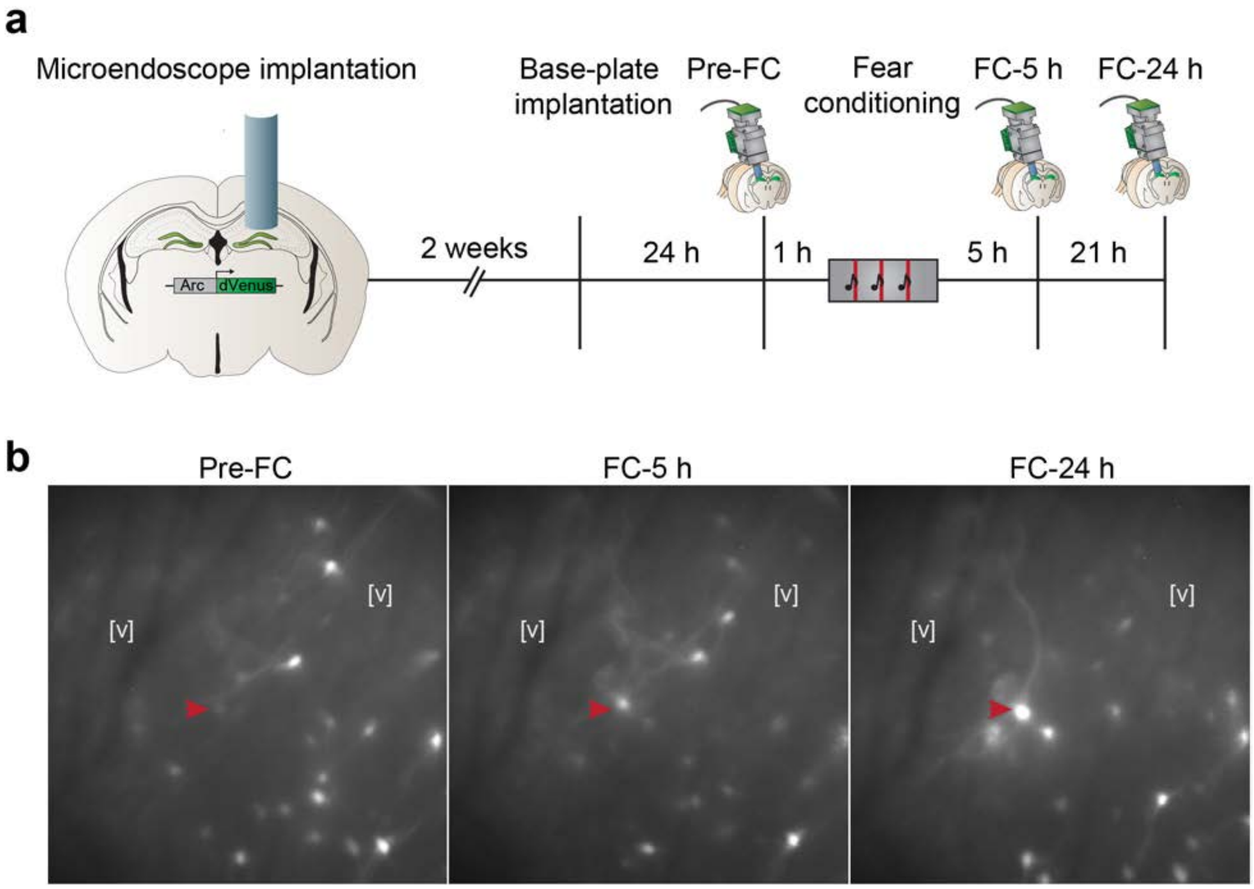

Supplementary Figure 14. *In vivo* microendoscopic imaging of *Arc*::dVenus^{+} neurons in the dentate gyrus of fear conditioned mice

(a) Experimental setup where *Arc*::dVenus mice were implanted with microendoscope GRIN lenses targeting the DG. Two weeks later base-plates were implanted after adjusting the field of view based on the presence of cells and/or landmarks such as veins. The DG was then imaged 1 h prior to (Pre-FC), as well as 5 h (FC-5 h) and 24 h (FC-24 h) after fear-conditioning.

(b) Representative images of the DG at the aforementioned time-points. Red arrow indicates an example of a granule cell that exhibits an increase in *Arc*::dVenus in the hours following conditioning. [v] indicates vascular landmarks that were used to temporally co-register the images.

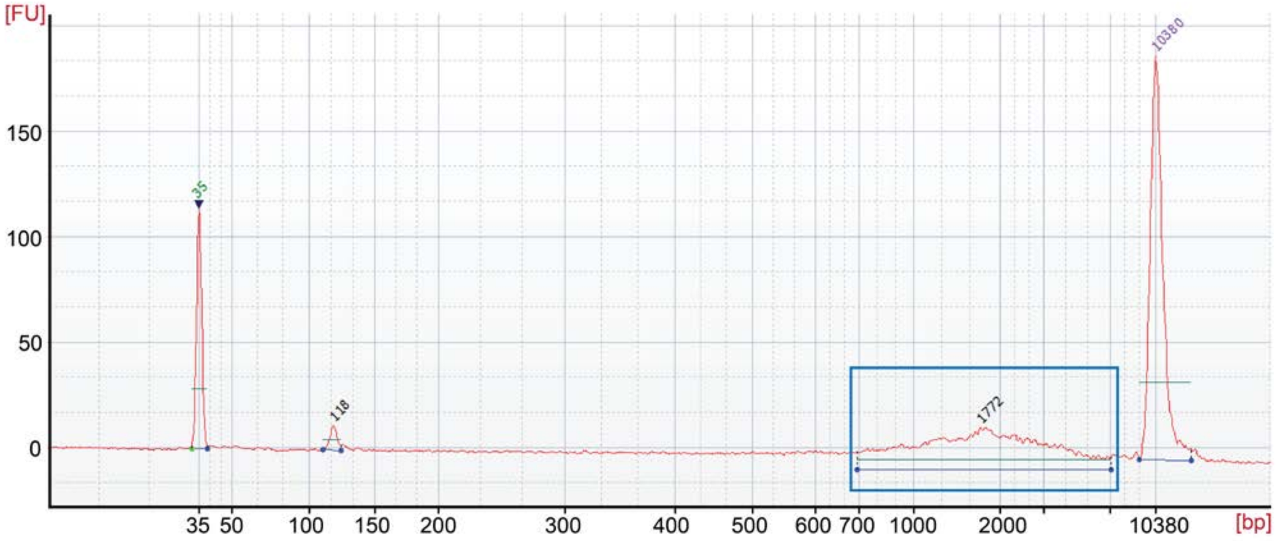

Supplementary Figure 15. Bioanalyzer data of cDNA quality control prior to amplification

A high sensitivity DNA assay was performed using an Agilent Bioanalyzer to measure cDNA quality and quantity prior to amplification[13]. Representative image of cDNA that was diluted 6 times, from 10 pooled DG granule cells showing that a majority of fragments (blue box) in the library have an average size larger than ≈1 kbp (blue box). Upper (purple, 10380bp) and lower (green, 35bp) peaks of the ladder are indicated. [FU]: fluorescence units, [bp]: base pairs.
