# Supplementary material for: Engram-specific transcriptome profiling of contextual memory consolidation
Source: Nat Commun. 2019 May 20;10:2232. doi: 10.1038/s41467-019-09960-x (PMC6527697; doi:10.1038/s41467-019-09960-x)
Supplement: Supplementary file 3 — Reporting Summary [file 41467_2019_9960_MOESM3_ESM.pdf]

nature research

Corresponding author(s): Kushner, S.A.; van den Oever, M.C.

Life Sciences Reporting Summary

Nature Research wishes to improve the reproducibility of the work that we publish. This form is intended for publication with all accepted life science papers and provides structure for consistency and transparency in reporting. Every life science submission will use this form; some list items might not apply to an individual manuscript, but all fields must be completed for clarity.

For further information on the points included in this form, see Reporting Life Sciences Research. For further information on Nature Research policies, including our data availability policy, see Authors & Referees and the Editorial Policy Checklist.

Please do not complete any field with "not applicable" or n/a. Refer to the help text for what text to use if an item is not relevant to your study. For final submission: please carefully check your responses for accuracy; you will not be able to make changes later.

Experimental design

1. Sample size

Describe how sample size was determined.	The number of animals in each experimental group are standard sample sizes used in fear conditioning and immunohistochemical counting studies

2. Data exclusions

Describe any data exclusions.	It was decided a priori that animals would be excluded only in case of misplaced viral injections. However, no animals were excluded as none met this criteria.

3. Replication

Describe the measures taken to verify the reproducibility of the experimental findings.	All immunohistochemical and behavior results were successfully replicated at least twice.<br>For RNA sequencing experiments, 3 independent replications were performed for the fear conditioned group, as noted in the methods section

4. Randomization

Describe how samples/organisms/participants were allocated into experimental groups.	Animals were age and weight-matched before being randomly assigned to either the home-cage, fear-conditioned or no-shock groups.<br>For mCREB behavior experiments, animals were randomly selected to receive either the mCREB or the control virus.

5. Blinding

Describe whether the investigators were blinded to group allocation during data collection and/or analysis.	For immunohistochemical and RNA scope experiments, the experimenter was blind to the experimental condition. All behavioral measurements of freezing were performed using automated systems.

Note: all in vivo studies must report how sample size was determined and whether blinding and randomization were used.

6. Statistical parameters

For all figures and tables that use statistical methods, confirm that the following items are present in relevant figure legends (or in the Methods section if additional space is needed).

n/a	Confirmed	
☐	☒	The exact sample size (*n*) for each experimental group/condition, given as a discrete number and unit of measurement (animals, litters, cultures, etc.)
☐	☒	A description of how samples were collected, noting whether measurements were taken from distinct samples or whether the same sample was measured repeatedly
☐	☒	A statement indicating how many times each experiment was replicated
☐	☒	The statistical test(s) used and whether they are one- or two-sided<br>*Only common tests should be described solely by name; describe more complex techniques in the Methods section.*
☐	☒	A description of any assumptions or corrections, such as an adjustment for multiple comparisons
☐	☒	Test values indicating whether an effect is present<br>*Provide confidence intervals or give results of significance tests (e.g. P values) as exact values whenever appropriate and with effect sizes noted.*
☐	☒	A clear description of statistics including central tendency (e.g. median, mean) and variation (e.g. standard deviation, interquartile range)
☐	☒	Clearly defined error bars in all relevant figure captions (with explicit mention of central tendency and variation)

See the web collection on statistics for biologists for further resources and guidance.

Software

Policy information about availability of computer code

7. Software

Describe the software used to analyze the data in this study.	Immunohistochemical, in vivo imaging data and RNA scope data was analyzed using ImageJ.<br><br>Freezing during fear conditioning experiments was quantified by automated sscoring software (MedAssociates and EthovisionX, Noldus)<br><br>RNA sequencing data was analyzed using:<br>1) The DeSeq2 package in R<br>2) Panther- gene list analysis<br>3) Ingenuity Pathway Analysis (Qiagen, V01-06)<br><br>Spss and GraphPad Prism were used for statistical analysis and generation of plots.

For manuscripts utilizing custom algorithms or software that are central to the paper but not yet described in the published literature, software must be made available to editors and reviewers upon request. We strongly encourage code deposition in a community repository (e.g. GitHub). *Nature Methods* guidance for providing algorithms and software for publication provides further information on this topic.

Materials and reagents

Policy information about availability of materials

8. Materials availability

Indicate whether there are restrictions on availability of unique materials or if these materials are only available for distribution by a third party.	All materials are available from the manufactures or from the corresponding authors (listed in the Methods section)

9. Antibodies

Describe the antibodies used and how they were validated for use in the system under study (i.e. assay and species).	The concentration and manufacture details of all antibodies has been listed in the methods section.<br>Antibodies against Arc (1:200), Fos (1:500) and Atf3 (1:00) were selected based on their successful application in previously published studies (Gouty-Colomer, L.A. et. al., Molecular Psychiatry (2016), Kitamura, T. et. al., Neuron (2015), Science (2017), Mladinic, M. et.al., Stem Cell Research (2014)) and were tested prior to use to determine optimal concentrations.<br>Furthermore, tissue stained without primary antibody helped rule out the possibility of non-specific binding of secondary antibody to the tissue.

10. Eukaryotic cell lines

a. State the source of each eukaryotic cell line used.	N/A
b. Describe the method of cell line authentication used.	N/A
c. Report whether the cell lines were tested for mycoplasma contamination.	N/A
d. If any of the cell lines used are listed in the database of commonly misidentified cell lines maintained by ICLAC, provide a scientific rationale for their use.	N/A

Animals and human research participants

Policy information about studies involving animals; when reporting animal research, follow the ARRIVE guidelines

11. Description of research animals

Provide all relevant details on animals and/or animal-derived materials used in the study.	Adult (postnatal weeks 8-12, 20-25gms), male 1) Arc::dVenus, 2) Fos::tTa transgenic and 3) C57Bl6/J wild-type mice, were used in all experiments

Policy information about studies involving human research participants

12. Description of human research participants

Describe the covariate-relevant population characteristics of the human research participants.	N/A
