# Supplementary material for: Engram-specific transcriptome profiling of contextual memory consolidation
Source: Nat Commun. 2019 May 20;10:2232. doi: 10.1038/s41467-019-09960-x (PMC6527697; doi:10.1038/s41467-019-09960-x)
Supplement: Supplementary file 4 — Description of Additional Supplementary Files [file 41467_2019_9960_MOESM4_ESM.pdf]

Supplementary Data file titles / legends

Supplementary Data 1. Quality control for RNA-seq libraries

Sequencing, Alignment and expression count statistics of every sample prepped for RNA-sequencing. Samples that failed quality control or sequencing quality control are indicated. For each library the number of detected genes are presented in the table. A gene is considered 'detected' if at least 1 fragments aligns to it. Genes are included in the differential expression analysis if they have a count >=5 in at least 4 samples for HC and NS, and a count >=5 in at least 8 samples for FC.

Supplementary Data 2. List of genes contributing to sample-sample principal component analysis.

Supplementary Data 3. Differential gene expression analysis

Differential expression analysis of all genes with an FDR adjusted *P*-value<0.05 along with their base mean, log2 fold change, log fold standard error, WALD statistics, *P*-value and FDR adjusted *P*-value is provided for the home-cage (HC) group in sheet 1, no-shock (NS) group in sheet 2, and fear conditioned (FC) group in sheet 3. The immediate early gene *Arc* has been highlighted in blue for each group.

Supplementary Data 4. Comparison to previously published data sets.

Comparison of HC, NS and FC data sets to previously published transcriptome analyses, based on significantly regulated genes (FDR corrected *P*-value<0.05 with absolute $\log_2$ fold change > 1.0) with corresponding heat maps.

1) Hermey G, Mahlke C, Gutzmann JJ, Schreiber J, Bluthgen N, Kuhl D. Genome-wide profiling of the activity-dependent hippocampal transcriptome. *PLoS One* 8, e76903 (2013): 2 data sets, 24 h and 1 h after seizure induction, whole hippocampus.

2) Lacar B, *et al*. Nuclear RNA-seq of single neurons reveals molecular signatures of activation. *Nat Commun* **7**, 11022 (2016): 1 data set, 1 h after novelty exposure, Fos+ neurons from the DG

3) Cho J, *et al*. Multiple repressive mechanisms in the hippocampus during memory formation. *Science* 350, 82-87 (2015): 4 data sets, 5 min, 10 min, 30 min and 4 h after contextual fear conditioning, whole hippocampus.

4) Cho JH, Huang BS, Gray JM. RNA sequencing from neural ensembles activated during fear conditioning in the mouse temporal association cortex. *Sci Rep* 6, 31753 (2016): 1 data set, 6 h after auditory fear conditioning, temporal association cortex (TeA), dVenus+ cells (pooled from FC and shock only control) versus whole TeA.

Every column indicates 1 data set and the log2 fold change of overlapping genes is listed.

Supplementary Data 5. List of enriched genes in the pathway analysis described in Fig. 2e and Supplementary Fig. 10.

Supplementary Data 6. List of upstream regulators with significant overlap *P* values for the FC data set.
